# Genomic insights into natural selection in the common loon (*Gavia immer*): evidence for aquatic adaptation

**DOI:** 10.1186/s12862-018-1181-6

**Published:** 2018-04-27

**Authors:** Zach G. Gayk, Diana Le Duc, Jeffrey Horn, Alec R. Lindsay

**Affiliations:** 1 Biology Department, Northern Michigan University, 1401 Presque Isle Avenue, Marquette, 49950 Michigan USA; 2 Biology Department, University of Windsor, 401 Sunset Avenue, Windsor, N9B 3P4 Ontario Canada; 3 Institute of Human Genetics, University of Leipzig Hospitals and Clinics, Leipzig, Germany; 4 Department of Evolutionary Genetics, Max Planck Institute for Evolutionary Anthropology, Leipzig, Germany; 5 Department of Mathematics and Computer Science, Northern Michigan University, 1401 Presque Isle Avenue, Marquette, 49950 Michigan USA

**Keywords:** Comparative genomics, Freshwater adaptation, Diving, Positive selection, Migration

## Abstract

**Background:**

The common loon (*Gavia immer*) is one of five species that comprise the avian order Gaviiformes. Loons are specialized divers, reaching depths up to 60 m while staying submerged for intervals up to three minutes. In this study we used comparative genomics to investigate the genetic basis of the common loon adaptations to its ecological niche. We used Illumina short read DNA sequence data from a female bird to produce a draft assembly of the common loon (*Gavia immer*) genome.

**Results:**

We identified 14,169 common loon genes, which based on well-resolved avian genomes, represent approximately 80.7% of common loon genes. Evolutionary analyses between common loon and Adelie penguin (*Pygoscelis adeliae*), red-throated loon (*Gavia stellata*), chicken (*Gallus gallus*), northern fulmar (*Fulmarus glacialis*), and rock pigeon (*Columba livia*) show 164 positively selected genes in common and red-throated loons. These genes were enriched for a number of protein classes, including those involved in muscle tissue development, immunoglobulin function, hemoglobin iron binding, G-protein coupled receptors, and ATP metabolism.

**Conclusions:**

Signatures of positive selection in these areas suggest the genus *Gavia* may have adapted for underwater diving by modulating their oxidative and metabolic pathways. While more research is required, these adaptations likely result in (1) compensations in oxygen respiration and energetic metabolism, (2) low-light visual acuity, and (3) elevated solute exchange. This work represents the first effort to understand the genomic adaptations of the common loon as well as other *Gavia* and may have implications for subsequent studies that target particular genes for loon population genetic, ecological or conservation studies.

**Electronic supplementary material:**

The online version of this article (10.1186/s12862-018-1181-6) contains supplementary material, which is available to authorized users.

## Background

Loons, order Gaviiformes, are a small extant group of five species, all of which are specialized for diving in freshwater and marine-coastal aquatic habitats. They exhibit a number of specialized morphological traits used for diving and piscivory, including posteriorly-positioned feet for foot-propelled diving, dense bones [1], and compressible feathers to reduce buoyancy forces [2]. Presumably many of these specialized anatomical and physiological traits are influenced by genes that have been subject to natural selection in loons and their ancestors.

Work from penguins [3] suggested that these ocean diving birds have been subject to positive selection in adipocyte, feather keratin, wing development, and opsin genes. In exclusively saltwater penguins (Sphenisciformes), genes controlling the shape of the eye lens were under selection to maximize sight below water [3]. In penguins, evidence for expansions of gene families related to lipid and beta-keratin production was found associated with polar saltwater aquatic foraging [3], but no prior work has focused purely on adaptations for freshwater aquatic diving. However, adaptation to freshwater aquatic habitats such as those occupied by loons during nesting, likely presents different selection pressures to saltwater aquatic divers such as penguins. First, freshwater habitats cut off from ocean currents freeze during winter, requiring long-distance migration biannually. Because optimal aerodynamics required for migration is at odds with the optimal morphology for diving, selection must balance both aquatic and flight morphologies in loons, unlike in flightless penguins where selection pressures are optimized for diving and terrestrial locomotion. This could result in selection on development genes as well as for increased lipid metabolism during migration.

Although loons are volant, they are unable to walk on land and are thus less terrestrial than flightless penguins [4]; loons only set foot to land to copulate and incubate eggs [4]. Loons are also required to osmoregulate in more varied conditions than most other aquatic birds, as loons spend roughly half of each year in saltwater habitats (non-breeding season) [5] and half of each year on freshwater habitats (breeding season) [4]. Comparing the origin of osmoregulatory adaptations in a phylogenetic framework is particularly worthwhile because of the recent advances in avian genomics which now allow for comparisons of species from nearly every avian order, including species of waterbirds that are either saltwater or freshwater specialists [6].

We sequenced and assembled a draft genome of *Gavia immer*, the common loon, to improve our understanding of how genomic features evolve during adaptation to aquatic habitats and diving. We performed comparative genomics analyses using red-throated loon genes along with common loon genes to increase the power to detect *Gavia-*specific positive selection, in relation to Adelie penguin, (*Pygoscelis adeliae*), chicken (*Gallus gallus*), northern fulmar (*Fulmarus glacialis*), and rock pigeon (*Columba livia*) genomes [7]. We identified genomic changes in the common loon that modulate their oxidative and energetic metabolism, which may support their diving behaviour.

## Results

### Genome assembly

The Illumina 2000 runs used 8 kb inserts to generate 100 bp paired-end reads. We only used reads that passed quality control filtering with scores between Q20 and Q30 (99.26% and 95.43% confidence in correct base call respectively). This reduced the initial number of 499,620,770 reads with 50,461,697,770 individual bases by 58.26%, leaving 291,098,878 usable reads totaling 26,946,081,239 individual bases post filtering.

ABySS assemblies of the common loon genome with eight different *k*-mer sizes ranging from *k*25—*k*64 yielded contig N50 values from 641 to 814 bp in length. The *k*-mer size that optimized contig N50 was *k* = 30 with an N50 of 814 bp. The *k* = 30 assembly was therefore selected as the best assembly to submit to evolutionary analyses. Based on BBMAP analyses, the *k* = 30 assembly consisted of 5,237,924 contigs with a total contig length of 767,326,331 bp. For the *k* = 30 assembly, *k*-mer coverage per ploidy of the diplod sequenced genome was estimated to be approximately 11.84×. Despite the fragmented nature of the genome assembly, 62,409 contigs had lengths of 1 kilobases (kb) or greater. While this comprised only 1.2% of the total contigs and 12.8% of total assembly length, such sequences were of sufficient length for analyses of entire genes and smaller sequences were still suitable for analyses of whole or partial exons.

**Fig. 1:**
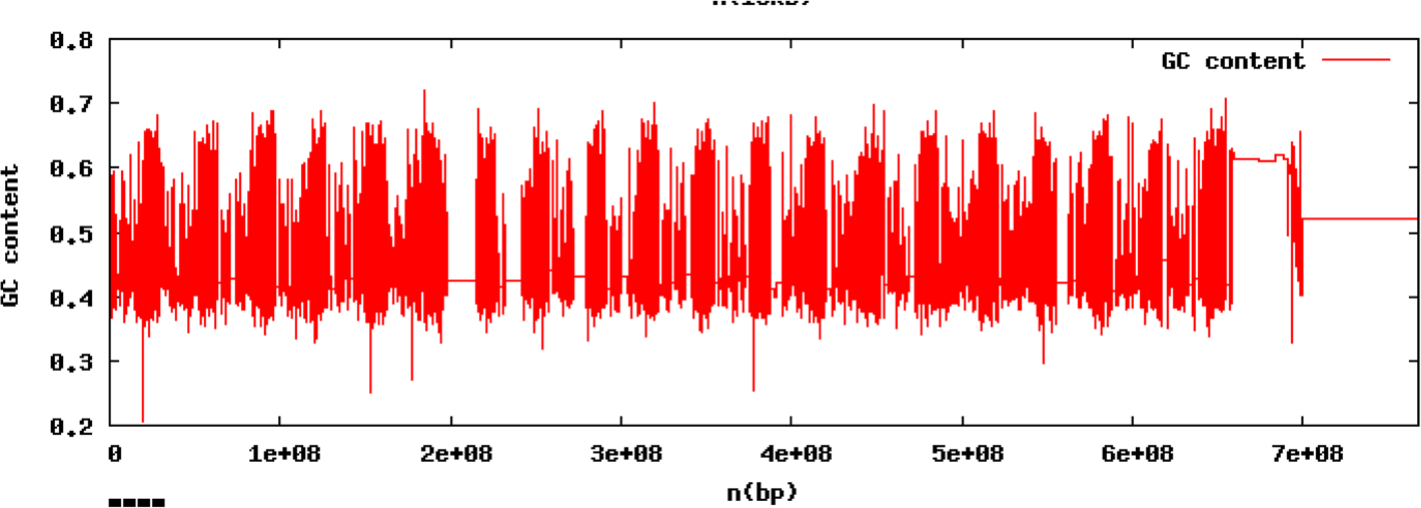
Percent GC content of the common loon *k* = 30 genome assembly. Variation in the percentage of bases composed of paired G (guanine) and C (cytosine) is shown across assembled bases from 1 to 767,326,331 base pairs in the total assembly length. Individual values of GC across the genome assembly were plotted using a sliding window analysis set to examine every 10 Kbp. Regions with no GC content, shown as white spaces, likely represent sequencing gaps

From calculations using BBMAP, percent GC content of the *k* = 30 assembly was estimated to be approximately 45.7%, while the proportion comprised of AT content was approximately 54%. Genomic GC content was highly heterogeneous across the genome assembly at a sliding widow size of 10 kb. Local variation in GC content ranged from approximately 30% to 70% (Fig. 1). GC content was especially low within the region spanning 2 Mbp of the genome assembly (Fig. 1), which most likely represents a difficult to assemble repetitive region of the common loon genome.

### Gene identification

From tabular BLAST output with *k* = 30 assembly scaffolds as query and chicken coding sequence as subject, a database of 136,755 matches to Ensembl transcripts [7] was returned. Ensembl transcripts had between two to eight result matches to the same scaffold in the *k* = 30 assembly. After filtering with BiomaRt [8], the list of genes identified in the *k* = 30 common loon assembly consisted of 13,821 known chicken genes in Ensembl release 81 and a further 348 unidentified transcripts with unknown function, for a total of 14,169 common loon genes (Additional file 1). These results indicate that 80.7% of chicken genes in Ensembl release 81 were identified within the common loon assembly (Additional file 2).

### Evolutionary analyses

Out of 14,169 common loon genes, a total of 9665 protein coding sequences were able to be frame-corrected and used in PAML analyses with a high degree of confidence (Additional files 1 and 3). The remaining 4504 fragments had no open reading frame despite aligning to known genes in the five other species.

**Fig. 2:**
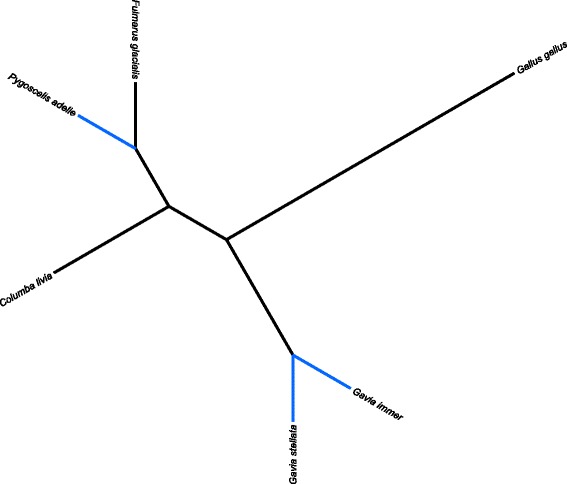
Cladogram representation of common loon (*Gavia immer*), red-throated-loon (*Gavia stellata*), northern fulmar (*Fulmarus glacialis*), Adelie penguin (*Pygoscelis adelie*), rock pigeon, (*Columba livia*), and chicken (*Gallus gallus*). The aquatic diving lineages are highlighted in blue

After removal of sequences with no synonymous mutations, which can lead to spurious dN/dS calculation [9], 700 out of 9665 gene sequences (7.2%) had dN/dS greater than one. Likelihood ratio tests resulted in a significant improvement of likelihood scores under the model of positive selection for 164 of 700 genes (23.4%). This set of 164 genes therefore had statistical support for positive selection in common loon and red-throated loons, relative to four other background genomes (Fig. 2) and were considered the final set of positively selected genes. In this gene-set, 41 *Gavia* genes with the highest LRT values had functions in solute exchange and ATP metabolism (Tables 1 and 2). A Candidate gene *GNB1* potentially involved in low-light signal transduction was also identified as positively selected, as was *HMOX1*, which may have a role in oxygen respiration under conditions of dive-induced local hypoxia.

**Table 1:** Common (*Gavia immer*) and Red-throated loon (*Gavia stellata*) genes with the highest statistical support indicating either positive selection or purifying selection, indicated by faster or slower evolution under COL_Gst_trend, respectively. Common loon is abbreviated COL and Red-throated loon is abbreviated Gst

GeneID	seq_length	lnl__model 0	ω model 0	lnl_model 2	ω background	COL_Gst	LRT	COL_Gst_trend
EPN3	327	− 1152.7014	0.01707	− 1104,593	0.01782	0.01984	96.217	faster
ZBTB5	249	− 672.25763	0.20841	− 661,74,052	0.08824	1258	21,034	faster
ASB13	IS 9	− 433.96493	0.2276	− 423.50404	0.03184	0.93344	20.922	faster
IL4I1	771	− 2150,5564	0.11603	− 2142,9682	0.09269	0,45,843	15.176	faster
ZSWIM8	342	− 438,89,839	0.05848	− 431,37,865	0.01099	2.00206	15.04	faster
TUBB3	228	− 670.71185	0.00912	−665,15,958	0.01716	0.00017	11.105	slower
315,773	228	−821.91841	0.01812	− 816.56462	0.02302	0.00154	10.708	slower
FBX02	252	− 702.0616	0.51324	− 696.7442	0,62,797	0,0001	10.635	slower
SYT11	354	− 831.44038	0.0465	− 826.3884	0.03646	1,27,058	10.104	faster
cKir2.3	906	− 1761.5536	0.02201	− 1756.6812	0.00922	0.11328	9.7449	faster
SEMA5A	258	− 624.89875	0.28427	−620.04765	0.16011	0.91611	9.7022	faster
PNPLA6	210	− 651.71811	0.01012	−646.99188	0.00121	0.03078	9.4525	faster
CENPJ	399	− 985.68333	0.52198	− 980.97548	0.71507	0.11002	9.4157	slower
POLR3A	219	−452.46578	0.03422	− 447.92294	0.0001	0.1951	9.0857	faster
TBC1D8	204	− 648.48472	0.0926	− 640.07889	0.0001	3.96808	16.812	faster
C5RNP3	234	−755,24,789	0.05295	− 748.12504	0.0001	0.69521	14.246	faster
PHAX	246	− 550,29,897	0.10575	− 543,21,977	0.0197	0.32286	14.158	faster
PCD H17	267	− 501.13075	0.0589	− 494.61423	0.0001	0.28444	13.033	faster
NT5C1A	234	− 446,31,726	0.01154	− 441,0027	0.0001	0,2497	10,629	faster
PITX2	354	− 1137.3695	0.03826	−1132,3063	0,01375	0.79758	10.126	faster
LGR5	354	− 662.90136	0.07076	− 658,29,332	0.02553	0.54755	9.2161	faster
MON1A	705	− 1313.0707	0.00959	− 1308.521	0.00345	0.05225	9.0994	faster
KCNJ5	615	− 1150.454	0.02875	− 1145,9671	0,01487	0.34767	8,9738	faster
CDH7	234	−465.08493	0.12236	− 460.98436	0.0509	1.84634	8.2011	faster
SCN2A	708	− 1625.9186	0.06846	− 1621.8739	0.04567	0.17324	8.0892	faster

**Table 2:** Significantly evolving genes shared or with similar gene functions between aquatic Common and Red-throated loons (*Gavia* lineage) and Adelie Penguin (*Pygoscelis adelie*). Gene omega values of 999 indicate a lack of nonsynonymous mutations, but are retained where the other lineage contains nonsynonymous mutations

GENE	Branch	Function	seq_length	ω model 0	ω COL_Gst	LRT	COL Gst trend
ATAD2B	Gavia	ATPase family, AAA domain containing 2B	213	0.13786	0.53611	5.06462	faster
BMP6	P. adelaie	bone morphogenetic protein 6	75	0.01916	999	4.69446	faster
BAMBI	Gavia	BMP and activin membrane-bound inhibitor homolog	156	0.10285	0.41534	6.69316	faster
CALHM3	P. adelaie	calcium homeostasis modulator 3	246	0.2885	2.52871	7.65325	faster
CIB1	Gavia	calcium and integrin binding 1	93	0.02077	17.66026	4.15427	faster
CDHR1	P. adelaie	cadherin related family member 1	228	0.06232	0.24732	7.04434	faster
CDH7	Gavia	cadherin 7, type 2	234	0.12236	1.84634	8.20113	faster
CHEK2	P. adelaie/Gavia	checkpoint kinase 2	246	0.06661	0.64046	5.29991	faster
DDX11	P. adelaie	DEAD/H (Asp-Glu-Ala-Asp/His) box helicase 11	114	0.29558	1.1461	5.05993	faster
DDX55	Gavia	DEAD (Asp-Glu-Ala-Asp) box polypeptide 55	168	0.19828	999	8.64589	faster
MAT1A	P. adelaie/Gavia	methionine adenosyltransferase I, alpha	51	0.08453	1.8137	6.53492	faster
MRPL41	P. adelaie	mitochondrial ribosomal protein L41	93	0.05377	999	5.09515	faster
MRPS30	Gavia	mitochondrial ribosomal protein S30	105	0.07025	0.26428	5.5027	faster
RNF144B	P. adelaie	ring finger protein 144B	201	0.41768	999	7.94759	faster
RNF151	P. adelaie	ring finger protein 151	129	0.01272	0.10092	3.84739	faster
RNF122	Gavia	ring finger protein 122	111	3.11562	4.51569	4.85065	slower
RNF14	Gavia	ring finger protein 14	171	0.21056	0.0001	4.13336	slower
RNF150	Gavia	ring finger protein 150	111	0.07654	0.17727	5.04599	faster
SLC15A4	P. adelaie	solute carrier family 15 (oligopeptide transporter), member 4	165	0.06417	999	5.59949	faster
SLC22A4	P. adelaie	solute carrier family 22 (organic cation transporter), member 4	213	0.09995	999	3.88664	faster
SLC30A8	P. adelaie	solute carrier family 30 (zinc transporter), member 8	144	0.04012	999	4.49118	faster
SLC20A1	Gavia	solute carrier family 20 (phosphate transporter), member 1	63	0.02234	0.43825	6.31834	faster
SLC32A1	Gavia	solute carrier family 32 (GABA vesicular transporter), member 1	963	0.00303	0.04424	5.14974	faster
SLC4A8	Gavia	solute carrier family 4, sodium bicarbonate cotransporter, member 8	126	0.02901	0.12003	7.24001	faster
SLC5A12	Gavia	solute carrier family 5 (sodium/monocarboxylate cotransporter), member 12	138	0.4122	0.0001	5.3412	slower
TBC1D24	P. adelaie	TBC1 domain family member 24	306	0.05272	1.25765	10.2849	faster
TBC1D8	Gavia	TBC1 domain family, member 8 (with GRAM domain)	204	0.0926	3.96808	16.8117	faster
WDR31	P. adelaie/Gavia	WD repeat domain 31	162	0.20233	0.0001	4.99304	slower

## Discussion

### Assembly quality

The highly fragmented state of the current *k* = 30 genome assembly, judged by both number of contigs and contig N50, is less than that of highly vetted genomes such as chicken, and zebra finch (*Taeniopygia guttata*) [10]. Recently published genome assemblies from birds including the Hume’s ground tit (*Pseudopodoces humilis*) [11], golden eagle (*Aquila chrysaetos*) [12], and Adelie penguin [13] all have contig N50 values in the range of 19—164 Kbp, whereas the current common loon assembly has a contig N50 of 814 bp. Assembly quality most closely approximates that of the black grouse (*Tetrao tetrix*) draft assembly [14], which had a contig N50 value of 1238 bp. Despite the fragmented nature of the current common loon assembly, contig lengths as indicated by N50 appear to be adequate for identifying protein coding regions of genes [15]. However, lacking long contiguous scaffolds, the exact placement and order of particular contigs can not be determined and thus is likely inadequate for analyses of synteny [16].

Conclusions about assembly quality must be put in the proper perspective about the resolution desired in analyses. For opportunistic analyses of selection between closely related species making use of available contigs, this assembly has value. The fragmented nature of the best ABySS assembly can be attributed to several factors including: (1) large (8 kb) insert libraries of only one size used in assembly, (2) a short read size of 100 bp, and (3) low sequencing depth. *K*-mer coverage per ploidy of the diploid loon genome was calculated to be 11.83×, whereas target *k*-mer coverage should be in the range of 20–30× for a high-quality genome assembly (B. Bushnell pers. comm.). In this assembly, the odds of correctly assembling each read per ploidy are low given that actual coverage per ploidy is between one-half to one-third the target range for a good genome assembly (B. Bushnell pers. comm.) [17].

### Genome size and GC content

Avian genomes are between one-third to one-half the size of mammalian genomes. This may occur because flight imposes metabolic constraints limiting cell size and increasing the efficiency of cellular metabolism with a higher cell surface area [18]. Estimates from well studied bird genomes suggest typical avian genomes should be in the range of 1—1.5 Gbp with a maximum number of genes under 20,000 [12]. The total consecutive sequence length of the common loon genome assembly was 767 Mpb. Although assembly quality makes estimation of the actual common loon genome size impossible, this figure could indicate we have assembled between 50 and 76% of the common loon total genomic content using published genome sizes [12] as a reference.

### Evolutionary analyses and biological significance of genes under positive selection

Patterns of gene enrichment suggest that selection since the common loon—chicken split approximately 90 mya [6] has acted on candidate genes related to hemoglobin affinity for oxygen, solute exchange, immunoglobulin function related to immune defense, nervous system development and a number of molecular pathways related to DNA metabolic function, and G-receptor pathways potentially involved in low-light visual acuity.

A selection analysis of emperor (*Aptenodytes forsteri*) and Adelie penguin (*Pygoscelis adeliae*) genomes identified a number of positively selected genes related to Antarctic diving and cold tolerance, and vision in low-light environments [3]. They found: a greater number of β-keratin genes—which comprise 90% of mature feather barbs and barbules—than in any other bird species, a reduction in the number of opsin genes to three trichromatic classes as opposed to four found in most birds as an adaptation to low light environments, positive selection in *FASN* which encodes lipid metabolism and lipogenesis, and mutation of 17 genes associated with short limb and truncated dorsal morphology for flipper-based diving.

Li et al.’s [3] focus on identifying genes associated purely with penguin flightlessness and polar marine physiology provide the most phylogenetically similar but still somewhat restricted comparison to loons from an ecological point of view. Although loons (Gaviiformes) and penguins (Sphenisciformes) both may have originated in the Southern Hemisphere [19] (the exact origin is unresolved), loons have since evolved to breed and forage on freshwater ponds and lakes during summer and to reside and forage in marine environments during winter. Different buoyancy forces and osmotic exchange rates exist in freshwater and saltwater environments and loons are one of few migratory aquatic bird classes that exploit both during the same year, and potentially the same day. *Aptenodytes* penguins dive much deeper than loons, reaching depths over 300 m, so selection pressures on visual systems might be different in penguins which experience lower light conditions than shallower diving loons [20]. No loon species have polar distributions and while they do inhabit cold arctic and boreal regions, migration limits the need for the specialized feather and adipose tissue of penguins. It is likely that genes for solute exchange and osmoregulation are the most important class of positively selected genes in the *Gavia* lineage when compared across five other avian genomes. *SLC48A*, and *SLC20A1* in particular may have a role in maintaining ion and pH balance and are therefore candidate genes in the *Gavia* lineage for maintaining ion homeostasis.

In common loons, the high aerobic and metabolic costs of a physiology adapted foremost to deep-water diving, but also long distance (trans-continental) aerial migration indicate disparate selection pressures have shaped loon evolution. The optimal morphology for diving, (i.e. high mass, easily concealed wings, ventrally positioned feet) presents severe trade-offs for flight, as high mass and narrow wings make it difficult for loons to become airborne and require high flight speeds once aloft [4]. We hypothesize that a number of genes associated with ATP and metabolism may have been positively selected in common loons to maximize energy production in these environments.

## Conclusions

Most of the hypotheses for positive selection in loons remain speculative unless confirmed through additional studies. However, now that candidate positively selected genes are available, future work could examine the expression of these genes through RNA-sequencing [21]; in particular the mechanisms through which osmoregulation is balanced in salt and freshwater environments, low-light phototransduction is achieved, and oxygen saturation is maximized in flight and diving should be elucidated. The most compelling approach to interpret the adaptive context of common loon evolution would integrate high-throughput genomic data (as in this study), and established common loon natural history, with direct hypothesis driven tests. This work provides a reference set of common loon genes that can now serve as targets for more detailed follow-up work. In addition, this study demonstrates that high throughput genome assembly methods can be used inexpensively to identify coding regions of genes. As next generation sequencing (NGS) continues to become more common and whole or partial genomic data become available for large numbers of species, more studies may develop tools to harvest incomplete genome assemblies for evolutionary comparative analyses.

## Methods

### De novo assembly

We used Illumina short read 2 × 100 base pair data generated by Axeq Technologies Inc. from a single female common loon for a de novo assembly of the common loon genome. The final sequencing output resulted in 499,620,770 sequence reads, comprising 50,461,697,770 total bases in all summed reads. We assembled the genome using the raw short read sequence read data with ABySS [22], which performs particularly well with complex vertebrate genomes [23].

We used the message passing interface (MPI) version of ABySS 1.5.2 [22] on a Rocks compute cluster to assemble eight versions of the common loon genome, each with different *k*-mer values. The highest quality genome assembly, judged by contig N50, was then annotated in an effort to maximize the length of protein coding regions for evolutionary analyses [17]. For the best assembly, we evaluated genome assembly completeness of entire proteins coding regions with the Core Eukaryotic Genome Mapping Approach (CEGMA) [12].

### Reference-guided assembly

To further improve contig lengths, we aligned each scaffold in our assembly to the publically available red-throated loon (*Gavia stellata*) genome [13]. To align scaffolds in the ABySS assembly to the red-throated loon genome we used the Burrows Wheeler Aligner (BWA) package [24]. After mapping the ABySS contigs to the red-throated loon genome, we again assessed the N50 of reference-mapped contigs and scaffolds and if they had not improved in length compared to the de novo assembly we extracted the paired-end Next generation sequencing (NGS) sequence read files and aligned the raw sequence reads to the red-throated loon genome [14]. We then merged consensus sequences from the ABySS [22] assembly alignment with the NGS read alignment using the program SAMTools [25]. This resulted in extension of scaffolds suitable for Gene Identification and analysis [14]. We used BBMAP [26] to extract assembly statistics and convert the assembly into fasta format.

### Gene identification

We used local BLASTn [27] to search the resulting common loon genome assembly, scaffold by scaffold, against the Adelie penguin, red-throated loon, chicken northern fulmar and rock pigeon [13] coding sequences (Ensembl release 81) [7]. We generated BLASTn [27] results using a custom-formatted script for 12-column tabular output. Gene names were then retrieved for each hit in the BLAST tabular output by using the Ensembl BiomaRt web interface [8]. To check whether we retrieve the same genomic region, chicken genes were subsequently mapped back to common loon scaffolds using a custom Python script.

### Evolutionary analyses

We used the resulting gene set to identify genes within the common loon assembly where evolutionary changes occurred since divergence from Adelie penguin, chicken, northern fulmar, and rock pigeon [6, 7]. To increase the power to detect *Gavia*-specific positive selection, we used red-throated loon (*Gavia stellata*) genes along with common loon genes identified in this study as the foreground branch.

As Illumina sequencing is not selective as to which DNA strand is amplified [28], 50% of the gene fragments in the loon assembly were on non-coding rather than coding strands. All common loon gene fragments analyzed for evidence of selection were first converted to coding strands and then placed into an open reading frame using custom Python scripts (Additional file 2). Because some identified gene sequences were fragments too short for biologically meaningful analysis of codons in PAML analyses [29], only fragments with the longest uninterrupted run of at least 200 aligned bases in each multiple sequence alignment were kept. We scanned for differently evolving genes with the CODEML program under a branch model (model = 2, two ωs for foreground and background branches, respectively, vs. model = 0, one ω for all branches, compared via likelihood ratio test), [30]. We computed Likelihood Ratio tests (LRT) for each ω ratio under a null assumption of purely neutral evolution. The resulting likelihood scores were used to calculate an LRT statistic, − 2(lnL_null_-lnL_estimated_), which was then compared to a chi-square distribution with one degree of freedom and α = 0.05.

## Additional files

Additional file 1: Total Genes. (XLSX 5169 kb)

Additional file 2: Methods and Overview. Description of Computer Resources. Locations of Genomic Assembly Data and Scripts. (DOCX 1266 kb)

Additional file 3: Significantly_Evolving_Genes. (XLSX 92 kb)
