# Supplementary material for: Genomic insights into natural selection in the common loon (Gavia immer): evidence for aquatic adaptation
Source: BMC Evol Biol. 2018 Apr 27;18:64. doi: 10.1186/s12862-018-1181-6 (PMC5921391; doi:10.1186/s12862-018-1181-6)
Supplement: Supplementary file 2 — Methods and Overview. Description of Computer Resources. Locations of Genomic Assembly Data and Scripts. (DOCX 1266 kb) [file 12862_2018_1181_MOESM2_ESM.docx]

Supplementary Materials.

Appendix 1. Supplemental Methods and Overview

*De Novo Assembly Overview.*—Assembly of NGS genomes uses either the de Bruijn graph or overlap-layout consensus algorithms. De Bruijn graph-based assemblers are currently considered more advanced than overlap-layout-consensus assemblers and so are more commonly used for large genomes (Compeau et al. 2011). Genome assembly based on the de Bruijn graph algorithm starts by dividing short NGS sequence reads (roughly 60-150 base pairs) into smaller fragments called *k*-mers of length *k* (Baker 2012). Although *k* should be carefully chosen, the *k*-mer method is used to decrease the computational difficulty of assembling billions of read fragments (Alkan et al. 2011). All *k*-mers with overlap between the DNA sequence are then aligned based on the number of shared nucleotides, and the actual genome sequence can then be determined. In this process, all contiguous overlapping fragment reads are aligned and assembled into the largest possible contiguous sequences without gaps in the underlying nucleotides. Such sequences, called contigs, represent the main result output of a good genome assembly.

After DNA assembly has reached the stage where fragments consist of numerous contigs isolated by remaining gaps where the genome sequence is ambiguous, the process of scaffolding begins. Most assembler programs now incorporate techniques to scaffold DNA into “super-contigs”, or contigs joined to other contigs by gaps in the sequence read quality (Swain et al. 2012). Scaffolds are assembled using paired-end read data, which allows the length comprising the gap sequence to be estimated [32].

*Reference Guided Assembly Overview*.—WE improved the quality of the best assembly produced by ABySS [16] using a reference guided assembly approach. Reference-guided genome assembly can be carried out one of two ways, which WE will describe below. The first technique consists of producing a de novo assembly and then aligning these contigs to a reference genome [19]. In the second technique, raw sequence reads generated via NGS are aligned to a reference genome and then the contiguous read fragments are exported and used to produce a genome assembly [19]. In this research, WE proposed to use the first technique because, as above, ABySS was used to produce an initial de novo genome assembly. Reference –guided assembly can be particularly useful when there is not great confidence in a de novo assembly and time and energy costs necessary to improve the assembly preclude further sequencing. Particularly if the assembly results in short contigs or scaffolds and there is no time to re-sequence the taxon under study, reference-guided assembly may be useful to improve the genome assembly. A technical concern in this study is that source NGS data for this project was produced using only one read library size (8 kb). A number of read libraries constructed of different sized inserts improve the quality of a de novo assembly because this yields information on where the NGS reads were sequenced from and the likelihood of one fragment adjoining another across gaps in the scaffolding process [65]. Scaffolding will therefore be difficult in any de novo assembly generated from our data with only 8 kb libraries. A reference-guided assembly approach may rectify this problem by building consensus scaffolds based on the proximity of contigs mapped to a reference genome.

*Genome Annotation Overview.—*Gene annotation is a complex process, requiring many software programs to achieve a meaningful annotation. Because of this, annotation pipelines have now been developed that integrate all software components into single pipelines. The most common approach for genome annotation consists of using basic local alignment search tool algorithms (BLAST) to search genome databases for similar sequences present in the unannotated genome assembly. RNA and expressed sequenced tag (EST) information can also be used in some programs. Before using BLAST searches, several annotation pipelines now incorporate programs to analyze regions of the assembled genome for repeat sequences arising from transposable elements. One such program, Repeat Masker, masks repeat regions in the genome assembly so that BLAST searches do not identify chimeric coding sequences within this region [66]. Subsequently, all potential coding regions are identified using BLAST searches to align with reference genomes. Then, several programs such as SNAP and AUGUSTUS can be used to make *ab initio* gene predictions for remaining regions of the genome that may include exons not otherwise identified using available reference genomes. The advantage of using an annotation pipeline software, such as MAKER2 is that once set to examine a specific genome assembly, the annotation process can be semi-automated. This significantly decreases the time spent otherwise manually annotating genome assemblies. To vouchsafe automated annotation settings and ensure they are giving accurate results, many annotations now include a manual review of aspects of the genome assembly. Proposed annotation methods included using MAKER2 (Holt and Yandell 2011). MAKER2 incorporates four separate programs to build a pipeline for annotation (Doyle et al. 2014) including Repeat-Masker, BLAST, AUGUSTUS and SNAP. First, Repeat Masker was used to identify regions of sequence repeats in the assembly, including those arising from LINE, and SINE repeats. However, problems implementing the MPI (message passing interface) version of MAKER limited our work to only identifying repeat regions of the common loon genome. WE had planned to use MAKER2 to initiate BLAST searches based on RNA, and protein data from other available bird genomes. The programs AUGUSTUS and SNAP were to be used to make *ab initio* predictions of gene function for remaining unidentified genes.

Coding Strand Retrieval.—WE identified template sequences for each gene by subtracting chicken subject start position from chicken subject end position for all BLASTn results using a custom python script. For those blast results with positive start minus end positions, this indicated that the sequenced DNA strand was the template, and the reverse compliment had to be retrieved to get the translated sequence. WE reverse complimented 6555 sequences for which WE only had the template strand out of 13,211 common loon sequences that met length criteria using a custom python script. Sequences on reverse versus forward strands were then sorted in Microsoft Excel to retain order. After coding sequences were obtained for all 13,211genes, WE implemented an approach to check and adjust the reading frame where required. This was necessary because BLAST-identified [23] genes were fragments rather than entire coding sequences of mRNA transcripts. Thus, returned alignments of common loon and chicken gene fragments were created by BLAST [23] to optimize *E*-score based on nucleotide rather than codon alignment and could therefore start with nucleotides from incomplete codons. As such bases shift the reading frame, WE used a custom python script to identify and correct open reading frames. First, WE searched each nucleotide-aligned ortholog pair of common loon and chicken for multiple stop codons or stop codons in the middle of the sequence. Only modifying sequences that met these criteria, WE iteratively added a maximum of two N bases to the beginning of each out of frame ortholog until stop codons disappeared. Frame adjustment removed 76 % of chimeric stop codons, but for the remaining 15% of sequences (3105) multiple stop codons remained. WE attributed these to alignment gaps inserted by BLAST that do not make biological sense. Sequences for which the frame could not be corrected using these modifications were removed from the analysis.

Appendix 2. Description of Computer Resources


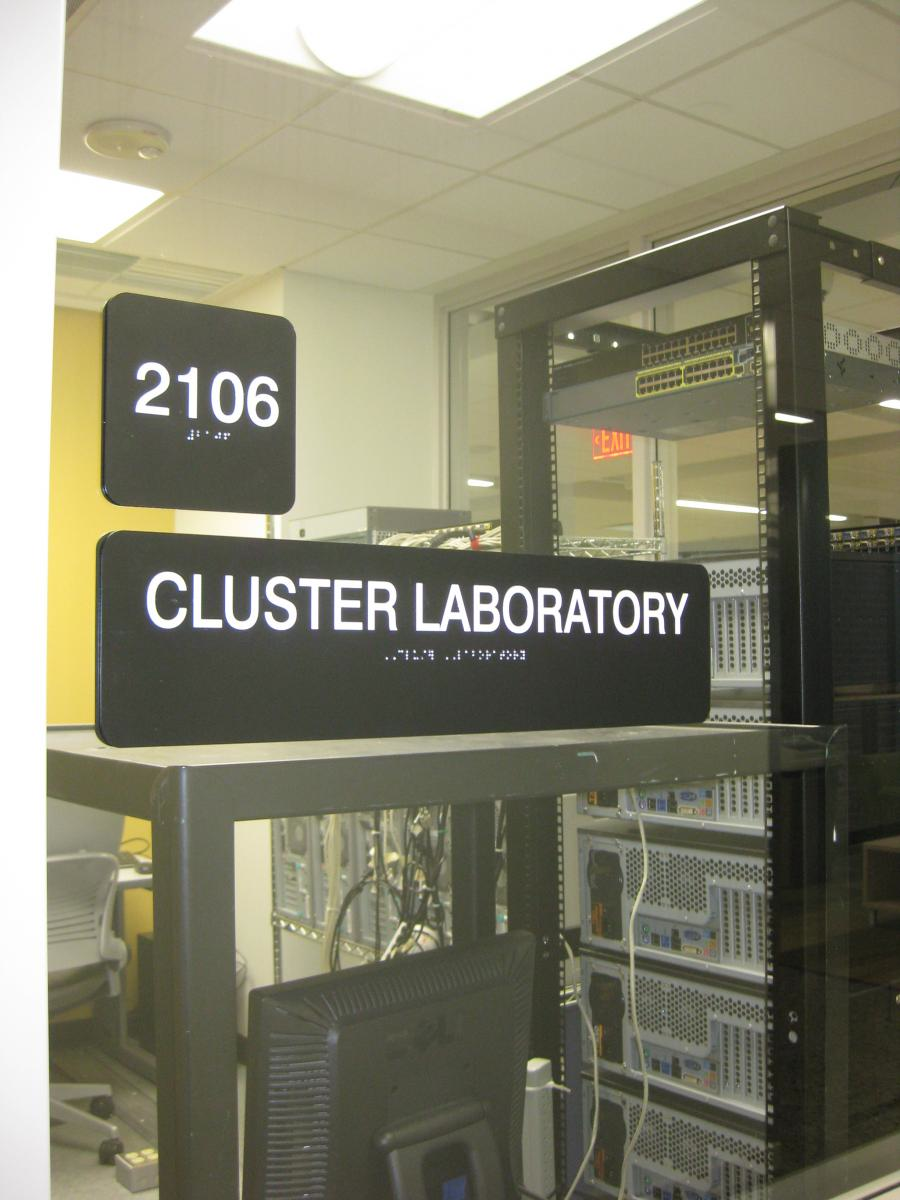


The following programs described in the content of the thesis above were run on a five-node compute cluster administrated by Dr. Jeff Horn:

ABySS- MPI

SAMTOOLS

BBMAP

BWA (SW)

PAML

The Williac cluster computer, located in the Cluster Laboratory of NMU's Mathematics

and Computer Science Department in room 2106 of Jarnrich Hall, consists of ten rack-mounted computers. All nodes host Intel Core i7 quad-core processors with sixteen gigabytes of RAM and solid state drives. They are interconnected by a gigabit per second Ethernet switch and run Rockscluster software on top of the centOS Linux operating system. The ABySS runs took approximately eight to ten hours using five of the compute nodes.

Appendix 3. Locations of Genomic Assembly Data and Scripts

There are three main directories of files generated from this research:

(1): Genome Assembly.—The fasta formatted genome assembly produced with ABySS [16] using *k* = 30 is located in the directory COLO_Assembly along with supplemental ABySS [16]files.

(2): Scripts and Manipulation.—All Python language environment scripts used for parsing and analyzing the common loon genome assembly are located in the directory Loon-Scripts; descriptions of the script function are given as comments (#) in the script itself.

(3): Gene Data.—All files produced during genome annotation and analysis, including lists of identified genes, Gene Ontology associations, and values from selection analyses are located in the directory COLO_Supplemental_Files. Supplemental file 1 consists of a list of all 13,821 identified genes in the common loon, Supplemental file 2 consists of a list of Gene Ontology categories for all these identified genes, and Supplemental file 3 consists of results from evolutionary analyses.
